# Supplementary material for: Light-dark dependent changes in chloroplast and mitochondrial activity in Chlamydomonas reinhardtii
Source: Front Plant Sci. 2025 Jul 17;16:1622214. doi: 10.3389/fpls.2025.1622214 (PMC12310610; doi:10.3389/fpls.2025.1622214)
Supplement: Supplementary file 1 [file DataSheet1.zip › Supplementary material/Supplementary Data.docx]

**Supplementary Data**

**Supplementary Figure S1: Phosphorylation levels of CrS6K protein. (A)** Western blot analysis of phosphoCrS6K and CrS6K protein was performed for the control cells and TOR kinase-inhibited cells (using AZD8055). The protein levels were normalized with respect to tubulin. **(B)** The ratio of phosphoCrS6K/CrS6K protein was quantified and plotted for both the control cells and TOR kinase inhibited cells. The data represents an average of two independent biological repeats.

**Supplementary Table S1: Quantification of cells in different mitochondrial morphologies in the light-dark cycle.** **(A)** Percentage of cells in different mitochondrial morphologies – tubular, intermediate, and fragmented. **(B)** Percentage of cells showing peripheral and diffused phenotype of mitochondria apposition with respect to chloroplast. All values represent mean ± SD, n=3.

**Supplementary Table S2: F (PSI/PSII) ratio in the light-dark cycle.** The ratio of fluorescence of PSI/PSII was quantified for all three conditions: **(A)** continuous light (LL), **(B)** continuous dark (DD), and **(C)** 12:12 hour synchrony (LD) across all the time points using wild-type cells. All values represent mean ± SD, n=3.

**Supplementary Table S3: Quantification of cells in different mitochondrial morphologies in the light-dark cycle upon TOR kinase inhibition. (A)** Percentage of cells in different mitochondrial morphologies – tubular, intermediate, and fragmented. **(B)** Percentage of cells showing peripheral and diffused phenotype of mitochondria apposition with respect to chloroplast. All values represent mean ± SD, n=3.

**Supplementary Table S4: F (PSI/PSII) ratio in the light-dark cycle in TOR kinase mutant.** The ratio of fluorescence of PSI/PSII was quantified for all three conditions: **(A)** continuous light (LL), **(B)** continuous dark (DD), and **(C)** 12:12 hour synchrony (LD) across all the time points using TOR kinase mutant. All values represent mean ± SD, n=3.


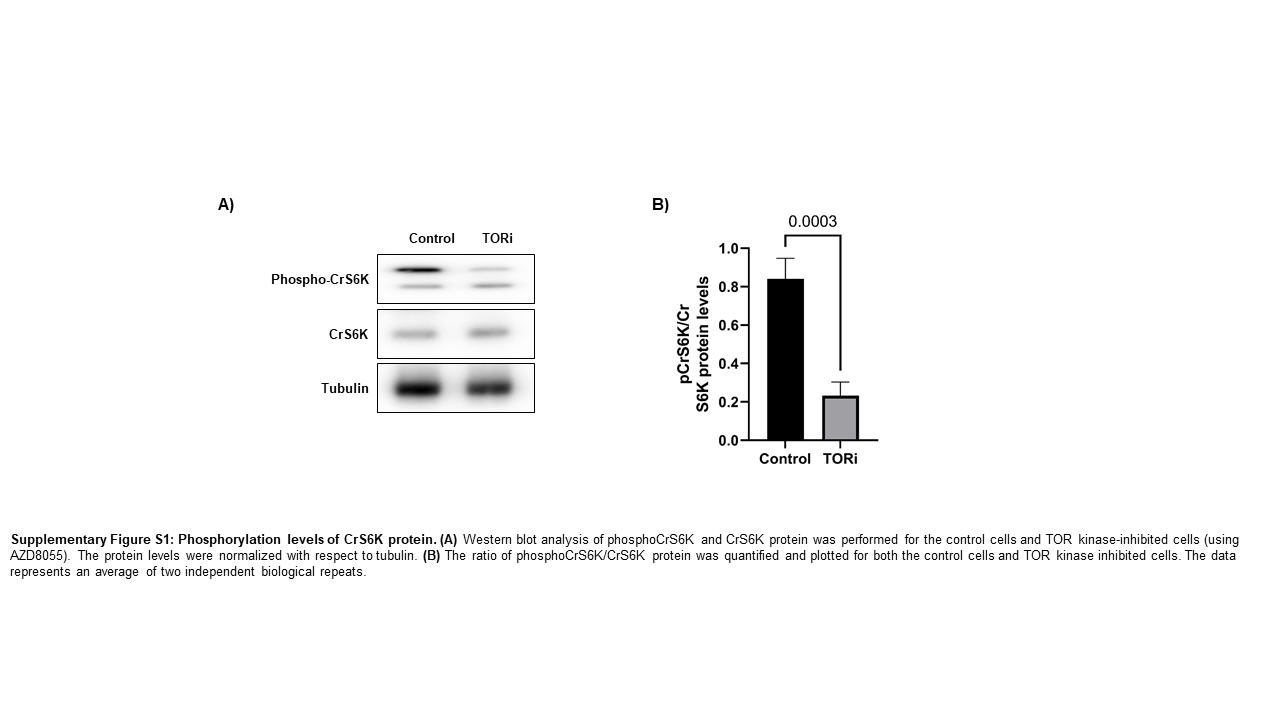


**1A.**

| **Control** | **0 hr** | **6 hr** | **12 hr** | **6 hr** | **12 hr** |
| --- | --- | --- | --- | --- | --- |
| **Tubular** | 9.91% ± 1.06% | 66.49% ± 3.76% | 81.46% ± 2.53% | 26% ± 1.16% | 3.55% ± 0.31% |
| **Intermediate** | 47.67% ± 1.4% | 25.95% ± 3.29% | 11.29% ± 1.68% | 26.08% ± 2.83% | 11.36% ± 1.66% |
| **Fragmented** | 42.42% ± 0.54% | 7.56% ± 0.78% | 7.25% ± 0.9% | 47.92% ± 3.12% | 86.27% ± 3.07% |

**1B.**

| **Control** | **0 hr** | **6 hr** | **12 hr** | **6 hr** | **12 hr** |
| --- | --- | --- | --- | --- | --- |
| **Peripheral** | 22.6% ± 1.94% | 68.45% ± 1.8% | 78% ± 0.47% | 33.08% ± 3.87% | 21.94% ± 1.30% |
| **Diffused** | 77.39% ± 1.94% | 31.55% ± 1.80% | 21.98% ± 0.47% | 66.92% ± 3.87% | 78.06% ± 1.3% |

**Supplementary Table S1: Quantification of cells in different mitochondrial morphologies in the light-dark cycle.** **(A)** Percentage of cells in different mitochondrial morphologies – tubular, intermediate, and fragmented. **(B)** Percentage of cells showing peripheral and diffused phenotype of mitochondria apposition with respect to chloroplast. All values represent mean ± SD, n=3.

| **A) Continuous light** | **0 hr** | **6 hr** | **12 hr** | **18 hr** | **24 hr** |
| --- | --- | --- | --- | --- | --- |
| **F(PSI/PSII)** | 1.37± 0.03 | 1.42 ± 0.08 | 1.32 ± 0.15 | 1.26 ± 0.06 | 1.4 ± 0.08 |
| **B) Continuous Dark** | **0 hr** | **6 hr** | **12 hr** | **18 hr** | **24 hr** |
| **F(PSI/PSII)** | 2.06 ± 0.07 | 1.78 ± 0.25 | 1.72 ± 0.05 | 1.86 ± 0.32 | 1.91 ± 0.14 |
| **C) Synchrony** | **0 hr** | **6 hr of light** | **12 hr of light** | **6 hr of dark** | **12 hr of dark** |
| **F(PSI/PSII)** | 1.2 ± 0.13 | 1.14 ± 0.03 | 0.91 ± 0.07 | 0.98 ± 0.1 | 1.2 ± 0.16 |

**Supplementary Table S2: F (PSI/PSII) ratio in the light-dark cycle.** The ratio of fluorescence of PSI/PSII was quantified for all three conditions: **(A)** continuous light (LL), **(B)** continuous dark (DD), and **(C)** 12:12 hour synchrony (LD) across all the time points using wild-type cells. All values represent mean ± SD, n=3.

| **TOR kinase inhibited cells** | **0 hr** | **6 hr** | **12 hr** | **6 hr** | **12 hr** |
| --- | --- | --- | --- | --- | --- |
| **Tubular** | 10.26% ± 1.67% | 63.68% ± 3.27% | 24.44% ± 1.99% | 3% ± 0.32% | 6.03% ± 2.59% |
| **Intermediate** | 42.22% ± 2.43% | 22.71% ± 1.63% | 34.37% ± 0.91% | 35.8% ± 0.44% | 38% ± 3.29% |
| **Fragmented** | 47.52% ± 1.4% | 13.6% ± 1.89% | 41.19% ± 2.85% | 61.2% ± 0.13% | 55.97% ± 3.24% |

**3A.**

**3B.**

| **TOR kinase inhibited cells** | **0 hr** | **6 hr** | **12 hr** | **6 hr** | **12 hr** |
| --- | --- | --- | --- | --- | --- |
| **Peripheral** | 8.38% ± 0.86% | 24.27% ± 1.26% | 18.64% ± 2.72% | 15.23% ± 5.83% | 7.21% ± 1.24% |
| **Diffused** | 91.62% ± 0.86% | 75.73% ± 1.26% | 81.36% ± 2.72% | 84.77% ± 5.83% | 92.79% ± 1.24% |

**Supplementary Table S3: Quantification of cells in different mitochondrial morphologies in the light-dark cycle upon TOR kinase inhibition. (A)** Percentage of cells in different mitochondrial morphologies – tubular, intermediate, and fragmented. **(B)** Percentage of cells showing peripheral and diffused phenotype of mitochondria apposition with respect to chloroplast. All values represent mean ± SD, n=3.

| **A) Continuous light** | **0 hr** | **6 hr** | **12 hr** | **18 hr** | **24 hr** |
| --- | --- | --- | --- | --- | --- |
| **F(PSI/PSII)** | 1.22 ± 0.03 | 1.44 ± 0.16 | 1.27 ± 0.14 | 1.35 ± 0.19 | 1.37 ± 0.27 |
| **B) Continuous Dark** | **0 hr** | **6 hr** | **12 hr** | **18 hr** | **24 hr** |
| **F(PSI/PSII)** | 1.92 ± 0.18 | 1.70 ± 0.23 | 1.74 ± 0.3 | 1.81 ± 0.31 | 2.11 ± 0.31 |
| **C) Synchrony** | **0 hr** | **6 hr of light** | **12 hr of light** | **6 hr of dark** | **12 hr of dark** |
| **F(PSI/PSII)** | 1.26 ± 0.15 | 1.12 ± 0.23 | 1.12 ± 0.07 | 1.13 ± 0.18 | 1.3 ± 0.32 |

**Supplementary Table S4: F (PSI/PSII) ratio in the light-dark cycle in TOR kinase mutant.** The ratio of fluorescence of PSI/PSII was quantified for all three conditions: **(A)** continuous light (LL), **(B)** continuous dark (DD), and **(C)** 12:12 hour synchrony (LD) across all the time points using TOR kinase mutant. All values represent mean ± SD, n=3.
