# Supplementary material for: Light-dark dependent changes in chloroplast and mitochondrial activity in Chlamydomonas reinhardtii
Source: Front Plant Sci. 2025 Jul 17;16:1622214. doi: 10.3389/fpls.2025.1622214 (PMC12310610; doi:10.3389/fpls.2025.1622214)
Supplement: Supplementary file 1 [file DataSheet1.zip › Supplementary material/Supplementary Video Legends.docx]

**Supplementary video legends**

**Supplementary Video S1: 3D representation of mitochondria and chloroplast morphology in continuous light condition.** Photoautotrophic *C. reinhardtii* mitochondrial GFP (MDH4-GFP) cells in continuous light were imaged using Leica TCS SP8 confocal laser scanning microscope and 3D projected.

**Supplementary Video S2: 3D representation of mitochondria and chloroplast morphology in continuous dark condition.** Photoautotrophic *C. reinhardtii* mitochondrial GFP (MDH4-GFP) cells in continuous dark were imaged using Leica TCS SP8 confocal laser scanning microscope and 3D projected.

**Supplementary Video S3A-S3E: 3D representation of mitochondria and chloroplast morphology in synchronous culture.** Photoautotrophic *C. reinhardtii* mitochondrial GFP (MDH4-GFP) cells in 12:12 hr light-dark cycle were imaged using Leica TCS SP8 confocal laser scanning microscope and 3D projected.

**Supplementary Video S4: 3D representation of peripheral positioning of mitochondria apposition to chloroplast cup in MDH4-GFP cells.**

**Supplementary Video S5: 3D representation of diffused phenotype of mitochondria apposition to chloroplast cup in MDH4-GFP cells.**

**Supplementary Video S6A-S6E: 3D representation of mitochondria and chloroplast morphology in TOR kinase inhibited synchronous culture.** Photoautotrophic *C. reinhardtii* mitochondrial GFP (MDH4-GFP) cells in 12:12 hr light-dark cycle were treated with TOR kinase inhibitor and imaged using Leica TCS SP8 confocal laser scanning microscope and 3D projected.

**Supplementary Video S7: 3D representation of peripheral positioning of mitochondria apposition to chloroplast cup in TOR kinase inhibited cells.**

**Supplementary Video S8: 3D representation of diffused phenotype of mitochondria apposition to chloroplast cup in TOR kinase inhibited cells.**
