# Supplementary figures and images for: Light-dark dependent changes in chloroplast and mitochondrial activity in Chlamydomonas reinhardtii
Source: Front Plant Sci. 2025 Jul 17;16:1622214. doi: 10.3389/fpls.2025.1622214 (PMC12310610; doi:10.3389/fpls.2025.1622214)

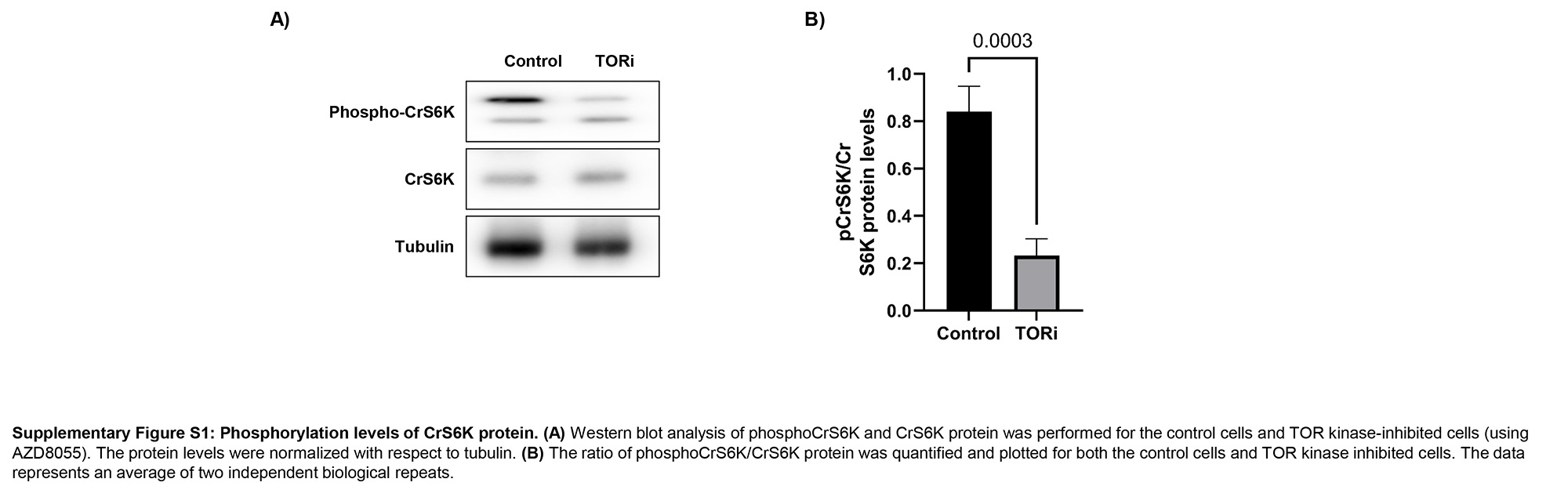

Supplement: Supplementary file 1 [file DataSheet1.zip › Supplementary material/Supplementary Figure 1.jpg]
